# Development of a Pre-Modification Strategy to Overcome Restriction–Modification Barriers and Enhance Genetic Engineering in *Lactococcus lactis* for Nisin Biosynthesis

**DOI:** 10.3390/ijms26052200

**Published:** 2025-02-28

**Authors:** Chen Chen, Yue Zhang, Ruiqi Chen, Ke Liu, Hao Wu, Jianjun Qiao, Qinggele Caiyin

**Affiliations:** 1 School of Chemical Engineering and Technology, Tianjin University, Tianjin 300072, China; cc_2019@tju.edu.cn (C.C.); 16602659785@163.com (Y.Z.); chenruiqi0803@163.com (R.C.); jianjunq@tju.edu.cn (J.Q.); 2 State Key Laboratory of Synthetic Biology, Tianjin University, Tianjin 300072, China; 3 Zhejiang Institute of Tianjin University, Shaoxing 312300, China; hllk@163.com

**Keywords:** *Lactococcus lactis*, restriction–modification system, gene manipulation, pre-modification system, nisin biosynthesis and regulation, nisin titer

## Abstract

Due to the barriers imposed by the restriction–modification (RM) system, Nisin-producing industrial strains of *Lactococcus lactis* often encounter low transformation efficiency, which seriously hinders the widespread application of genetic engineering in non-model *L. lactis*. Herein, we present a novel pre-modification strategy (PMS) coupled with optimized plasmid delivery systems designed to systematically evade RM barriers and substantially improve Nisin biosynthesis in *L. lactis*. Through the use of engineered *Escherichia coli* strains with methylation profiles specifically optimized for *L. lactis* C20, we have effectively evaded RM barriers, thereby facilitating the efficient introduction of large Nisin biosynthetic gene clusters into *L. lactis*. The PMS tools, which significantly improve the transformation efficiency (~10^3^ transformants per microgram of DNA), have been further improved in combination with a Rolling Circle Amplification, resulting in a higher enhancement in transformation efficiency (~10^4^ transformants per microgram of DNA). Using this strategy, large Nisin biosynthetic gene clusters and the expression regulation of all genes within the cluster were introduced and analyzed in *L. lactis*, leading to a highest Nisin titer of 11,052.9 IU/mL through a fed-batch fermentation in a 5 L bioreactor. This is the first systematic report on the expression regulation and application of a complete Nisin biosynthesis gene cluster in *L. lactis*. Taken together, our studies provide a versatile and efficient strategy for systematic evasion and enhancement of RM barriers and Nisin biosynthesis, thereby paving the way for genetic modification and metabolic engineering in *L. lactis*.

## 1. Introduction

*Lactococcus lactis* (*L. lactis*) is a prominent probiotic bacterium known for producing Nisin, a safe antimicrobial peptide widely used in the food industry [1,2,3,4]. Nisin is a naturally occurring lantibiotic produced by *L. lactis*, widely recognized for its potent antimicrobial properties, particularly against Gram-positive bacteria [5]. Due to its broad-spectrum efficacy, Nisin has found extensive applications in the food industry as a preservative, extending shelf life and preventing spoilage by pathogenic microorganisms [6]. Furthermore, its safety profile and low toxicity have led to its approval for use in both food and pharmaceutical applications. Beyond its antimicrobial effects, Nisin has been explored for potential therapeutic applications, including anticancer and immunomodulatory activities [7]. However, the efficient production and regulation of Nisin in industrial strains of *L. lactis* are often hindered by challenges, such as genetic manipulation barriers, primarily due to the activity of the restriction–modification (RM) system in these non-model organisms [8,9,10]. Thus, improving the genetic engineering of *L. lactis* to enhance Nisin production holds significant promise for advancing biotechnological applications in various sectors.

Regarding the improvement of the transformation efficiency in lactic acid bacteria, the current literature includes reports on optimizing electroporation conditions, such as electric field strength, pulse frequency, electric resistance, and pulse duration [8,11]. In addition, some studies focus on optimizing the preparation of competent cells, including factors such as growth stage, cell density, cell wall weakening agents, and buffer solution [9,12,13]. Due to the powerful restriction–modification (RM) system, optimizing electroporation parameters is not sufficient to achieve significant breakthroughs in improving transformation efficiency of *L. lactis*. Therefore, overcoming the RM system in *L. lactis* is a major challenge for widely utilizing its genetic manipulation and metabolic engineering.

The restriction–modification (R-M) system in bacteria plays a critical role in determining transformation efficiency, the ability of bacteria to uptake and express foreign DNA [14]. The RM system is a defense mechanism that protects bacterial cells from invading foreign DNA, such as plasmids or bacteriophage genomes, by employing two coordinated enzymatic activities, restriction endonucleases (REase) and DNA methyltransferases (MTase) [15,16,17]. When foreign DNA is introduced into a bacterial cell during transformation, it often lacks the specific methylation patterns of the host bacterium. As a result, the restriction enzymes recognize the unmethylated recognition sites on the foreign DNA and cleave it, leading to a significant reduction in transformation efficiency [18,19,20,21]. Among the current methods to overcome RM systems, bypassing plasmid DNA through restriction-negative, proficiently modified strains [22,23,24,25] and mimicking methylation profiles of certain strains [19,26,27,28,29,30] are the most effective approaches, which have been well validated in *Staphylococcus aureus* and *Rhodococcus ruber*. However, to date, there have been no reports on the development of tools for evading the RM systems in *L. lactis*.

**Figure 1:**
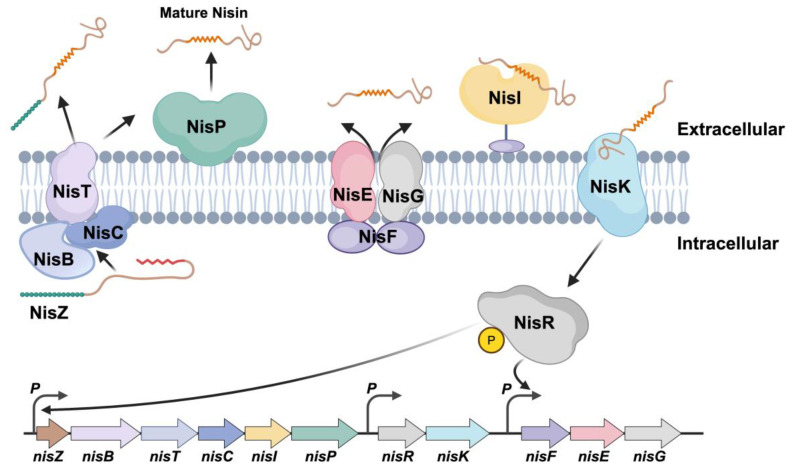
Biosynthesis and regulation mechanism of Nisin.

The biosynthesis of Nisin produced by *L. lactis* involves a complex enzymatic pathway that includes the modification of a precursor peptide to generate the mature active form (Figure 1). These enzymes catalyze the post-translational modifications, such as the formation of lanthionine rings, which are critical for Nisin’s antimicrobial activity. Over the past few decades, significant progress has been made in understanding the genetic and enzymatic mechanisms underlying Nisin production. However, the industrial application of Nisin production has been limited by low yields, primarily due to the inherent genetic manipulation barriers in *L. lactis*, particularly the activity of the restriction–modification (RM) systems. These systems impede the successful transformation of foreign DNA, making genetic engineering a challenge in non-model organisms such as *L. lactis*.

In this work, we report a pre-modification strategy (PMS) and optimized plasmid delivery systems to systematically evade the RM barrier and significantly enhance Nisin biosynthesis for the first time in *L. lactis*. *L. lactis* C20 is a non-model organism that inherently exhibits promising Nisin biosynthesis capability, demonstrating significant potential for industrial applications. This makes it an excellent candidate as a chassis cell for further development and optimization. However, the low plasmid transformation efficiency has impeded genetic manipulation of *L. lactis* C20. Consequently, through comprehensive analysis of genome and methylome, the RM systems with specific target recognition motifs were identified in *L. lactis* C20. By utilizing engineered *Escherichia coli* strains with specific methylation profiles tailored for *L. lactis* C20, we overcame the RM barriers, enabling the introduction of large Nisin biosynthetic gene clusters into *L. lactis* with high efficiency. These advancements not only enhanced the production of Nisin but also facilitated the regulation and optimization of the Nisin biosynthesis pathway, leading to higher Nisin titers. As the demand for Nisin continues to rise in the food and pharmaceutical industries, efficient genetic manipulation tools are critical to scaling up its production and optimizing its properties. Therefore, our study provides an effective strategy for systematic evasion and improvement of RM barriers and Nisin titer, expanding the possibilities of genetic modification and metabolic engineering in *L. lactis*.

## 2. Results

### 2.1. Identification of RM Systems in L. lactis C20

**Figure 2:**
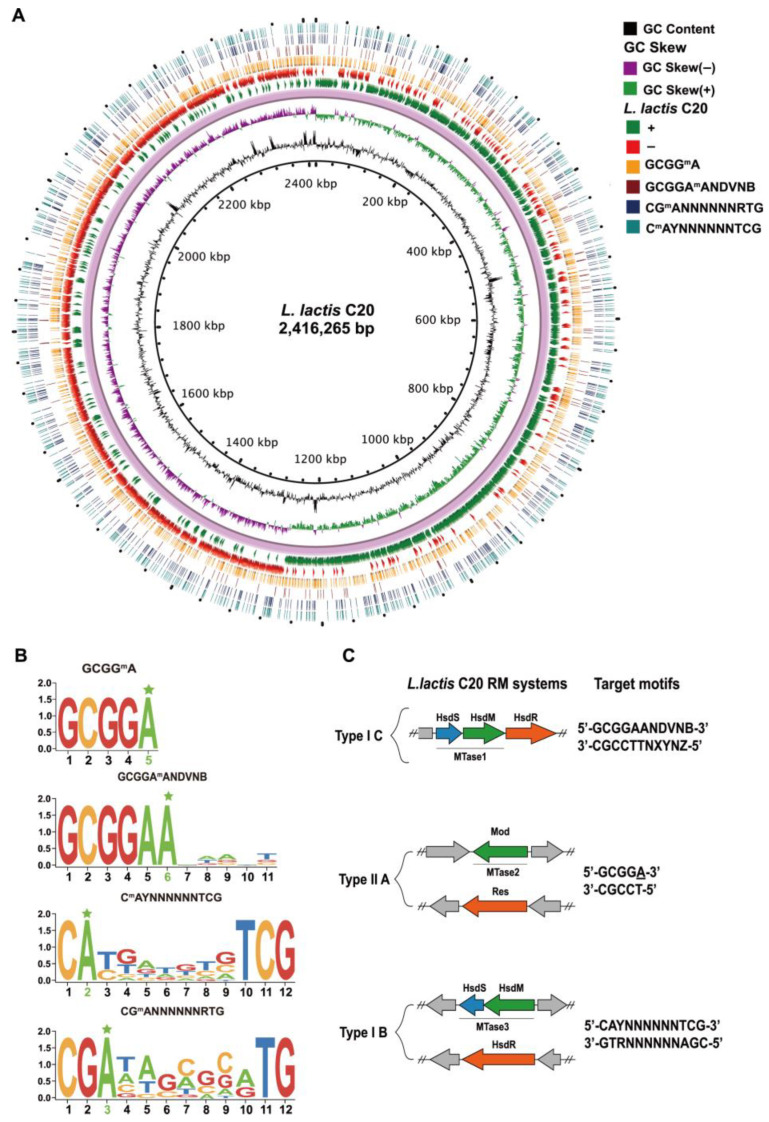
The methylome profile of *L. lactis* C20. (**A**) The genome profile, detected motifs, and methylated positions in *L. lactis* C20. The motifs are arranged according to modification type of the RM system and ordered from inner to outer circle, with their occurrence in genomes as shown in the plot legend. (**B**) MTase specificities are determined based on the detected methylated genomic positions. The degree of conservation (bits) is represented by the height of each stack, while the relative frequency of the base is represented by the height of the letters. The partner motifs C**A**YNNNNNNTCG and CG**A**NNNNNNRTG are both methylated on both strands, and all motifs are recognized by N-6 adenine-specific methyltransferases. The pentagram symbol in green represents the methylated bases. (**C**) Gene structure and recognition motifs of RM systems in *L. lactis* C20. The genes associated with the REase (Res and HsdR) and methyltransferases (HsdM and Mod) are represented in orange and green, respectively, while the genes encoding the specificity subunit (HsdS) are represented in blue.

The RM system of *L. lactis* C20, including REase and MTase genes as well as recognition motifs, was identified through SMRT-seq. The distribution of methylated motifs is random and evenly dispersed throughout the genome (Figure 2A), and the methylation profile of the RM system is summarized in Table S3. Three MTases were identified through motif analysis, including 5′-GCGG^m^6A-3′, 5′-C^m6^AYNNNNNNTCG-3′, 5′-CG^m6^ANNNNNNRTG-3′, and 5′-GCGGA^m6^ANDVNB-3′ (Figure 2B). In identifying motifs, the SMRT-seq analysis showed that motifs B1 and B2, which form a set of partner motifs, have methylated bases on both strands in all cases where they appear within the genome. It is worth noting that the methylation pattern was only detected on one strand in the case of motifs A and C. The degree of methylation was found to be nearly complete for the prominent methylation motifs A, B1, and B2, with methylation percentages ranging from 99.67% to 100%. In contrast, the m6A methylation motif C sequences exhibited a considerably lower level of methylation, with only 19.92% of the sequences displaying evidence of methylation. Three type I RM system motifs and one type II RM system motif were identified as being associated with the m6A methylation. Among these patterns, two were found to be palindromic in nature, resulting in methylation on both strands, while the remaining two may not be a perfect palindrome and were only hemi-methylated. Additionally, among the evaluated *L. lactis* C20, the most predominant m6A modification in terms of the number of methylated sites was observed to be 5′-GCGG^m6^A-3′, followed by 5′-C^m6^AYNNNNNNTCG-3′, 5′-CG^m6^ANNNNNNRTG-3′, and 5′-GCGGA^m6^ANDVNB-3′ (Table S3; Figure 2A). However, our analysis did not find any motif that could be attributed to the type III RM and type IV RM systems.

By gaining a deeper understanding of the presence or absence of RM-encoding genes in the strains, we can establish a correlation between the presence of specific methylated motifs with the existence of the RM systems in *L. lactis* C20. After identifying genes using REBASE, they were identified as two type I RM system MTases and one type II RM system MTase, referred to as MTase1, MTase2, and MTase3, respectively (Figure 2C). Therefore, we attempted to utilize these methyltransferases to construct PMS tools for evasion of the RM system in subsequent studies.

### 2.2. Development of PMS Tools and Evasion of RM Systems for High Transformation Efficiency

**Figure 3:**
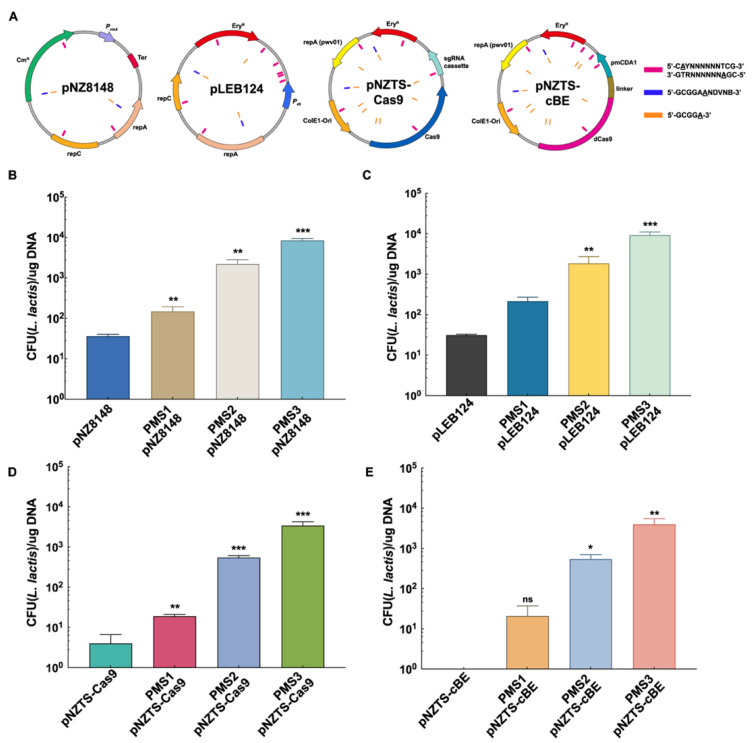
RM recognition sites and transformation efficiency of plasmids by PMS tools. (**A**) The distribution of RM system target recognition sites on plasmids pLEB124 (4.6 kb), pNZ8148 (3.2 kb), pNZTS-Cas9 (9.6 kb), and pNZTS-cBE (11.3 kb). The RM system target recognition motifs 5′-CAYNNNNNNTCG-3′, 5′-CGANNNNNNRTG-3′, 5′-GCGGAANDVNB-3′, and 5′-GCGGA-3′ are indicated as red, blue, and orange rectangles, respectively. (**B**) Transformation efficiency of pNZ8148 (CFU/μg DNA) by PMS1, PMS2, and PMS3 tools. (**C**) Transformation efficiency of pLEB124 (CFU/μg DNA) by PMS1, PMS2, and PMS3 tools. (**D**) Transformation efficiency of pNZTS-Cas9 (CFU/μg DNA) by PMS1, PMS2, and PMS3 tools. (**E**) Transformation efficiency of pNZTS-cBE (CFU/μg DNA) by PMS1, PMS2, and PMS3 tools. Data are shown as means ± SD from three biologically independent replicates. ns, no significance; *, *p* < 0.05; **, *p* < 0.01; ***, *p* < 0.001.

In three rounds of iterative genome editing, the engineered *E. coli* PMS1, PMS2, and PMS3 strains were obtained, each with a specific methylation pattern of *L. lactis* C20 (Figures S1, S2 and S7). Thus, the transformation efficiency of *E. coli*–*L. lactis* shuttle plasmids pNZ8148, pLEB124, pNZTS-Cas9, and pNZTS-cBE was determined, which were selected to represent distinct variations in terms of size and functionality. Within the diverse plasmids, we consistently observed the presence of all the RM target recognition motifs, and these motifs were found to be distributed evenly throughout the entire plasmid (Figure 3A). Therefore, PMS tools were utilized to pre-methylate these plasmids and analyze their transformation efficiency.

To investigate the transformation efficiency, the plasmids pre-modified with PMS1, PMS2, and PMS3 were transformed into *L. lactis* C20, and non-PMS plasmids were used as control. The existence of a functional methylation profile of MTase1 was confirmed by 12-fold, 6.9-fold, 29-fold, and 100-fold increases in transformation efficiency of pNZ8148, pLEB124, pNZTS-Cas9, and pNZTS-cBE, respectively (Figure 3B–E). Compared to PMS1, PMS2 showed higher transformation efficiency of all the plasmids, achieving ~5 × 10^2^ transformants per microgram DNA in transformation efficiency of large plasmids pNZTS-Cas9 and pNZTS-cBE. It is worth noting that when there is a complete RM methylation profile, PMS3 showed a considerable increase in transformation efficiency, particularly for large plasmids, increases of several orders of magnitude were achieved in pNZTS-Cas9 and pNZTS-cBE (~3 × 10^3^ transformants per microgram of DNA) (Figure 3D,E). Importantly, the PMS tools facilitated a breakthrough in the transformation of the pNZTS-cBE plasmid, transitioning it from non-transformation to efficient transformation. All PMS tools significantly improved the transformation efficiency. Interestingly, as the integrity of the methylation profile increases, the improvements in transformation efficiency become more pronounced. Therefore, the PMS3 tool was selected for further optimization due to its outstanding performance. These results clearly indicate that genome editing of the *E. coli* strains yielded a series of desired engineered strains, demonstrating the practicability of the PMS strategy, which aims to evade the RM systems through plasmid pre-modification.

### 2.3. Further Enhancing Transformation Efficiency Through PMS-RCA

Given that the literature suggests that RCA products have a positive effect on transformation efficiency across multiple cell types and homology lengths, we propose the idea of optimizing the PMS tool using the RCA method to further improve transformation efficiency [31]. Then, the RCA products of pre-modified PMS3 plasmids are transformed into *L. Lactis* to determine the transformation efficiency (Figure S5).

**Figure 4:**
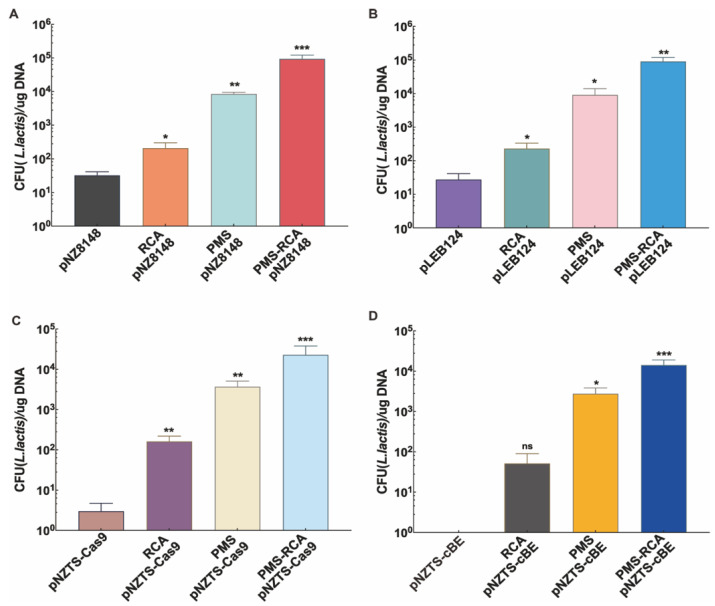
Transformation efficiency of plasmids by PMS-RCA tools. (**A**) Transformation efficiency of p NZ8148 (CFU/μg DNA) by RCA, PMS, and PMS-RCA tools. (**B**) Transformation efficiency of pLEB24 (CFU/μg DNA) by RCA, PMS, and PMS-RCA tools. (**C**) Transformation efficiency of pNZTS-Cas9 (CFU/μg DNA) by RCA, PMS, and PMS-RCA tools. (**D**) Transformation efficiency of pNZTS-cBE (CFU/μg DNA) by RCA, PMS, and PMS-RCA tools. Data are shown as means ± SD from three biologically independent replicates. ns, no significance; *, *p* < 0.05; **, *p* < 0.01; ***, *p* < 0.001.

For plasmid pNZ8148, PMS-RCA has the highest transformation efficiency, followed by PMS and RCA tools for *L. Lactis* (Figure 4A and Figure S6A–C). Similarly, pLEB124 showed the highest transformation efficiency using PMS-RCA (~10^4^ transformants per microgram of DNA s), an increase of ~3000-fold compared to the original unmodified plasmids (Figure 4B and Figure S6D–F). In the case of the plasmid pNZTS-Cas9, significant enhancements in transformation efficiency were observed using RCA, PMS, and PMS-RCA tools. After binding pNZTS-Cas9 to RCA, ~10^2^ transformants per microgram of plasmids was achieved in transformation efficiency, while PMS-RCA achieved ~2 × 10^4^ transformants per microgram of DNA, representing a further ~200-fold increase in transformation efficiency over RCA (Figure 4C and Figure S6G–I). Compared with other plasmids, pNZTS-cBE showed the highest improvement of transformation efficiency among all experimental groups. Using PMS-RCA, the transformation efficiency of pNZTS-cBE reached up to ~2 × 10^4^ transformants per microgram of DNA (Figure 4D and Figure S6J–L). This indicates that PMS-RCA could further enhance the transformation efficiency of the large plasmids in *L. Lactis*.

### 2.4. Enhancing Nisin Titers by Introducing Large Nisin Biosynthetic Gene Cluster

**Figure 5:**
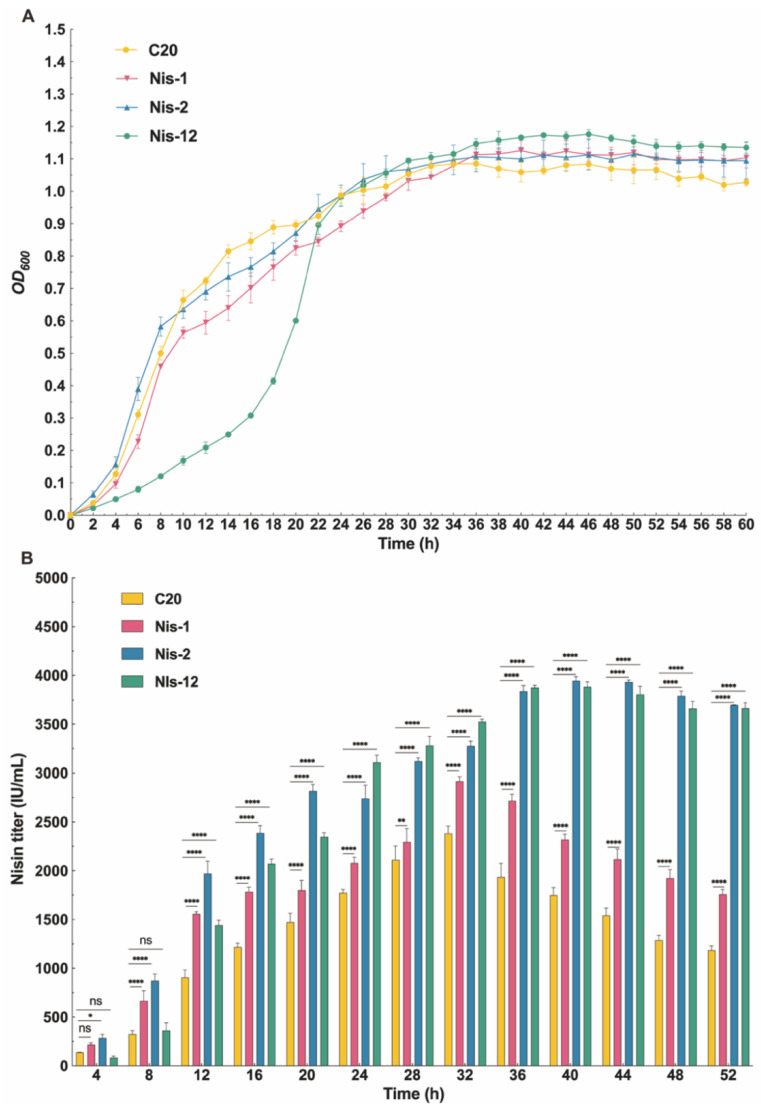
Growth profiles and Nisin titers of engineered *L. lactis* strains. (**A**) Growth curves at different time points. (**B**) Determination of Nisin titers at different time points. C20, the original strain *L. lactis* C20, as control group; Nis-1, the engineered strain *L. lactis* Nis-1 harboring pNZ8148-Nis1; Nis2, the engineered strain *L. lactis* Nis2 harboring pLEB124-Nis2; Nis-12, the engineered strain *L. lactis* Nis-12 harboring dual-plasmid system with pNZ8148-Nis1 and pLEB124-Nis2. Data are presented as means ± SD from three parallel replicates. ns, no significance; *, *p* < 0.05; **, *p* < 0.01; ****, *p* < 0.0001.

Using this tool, the large Nisin biosynthetic gene cluster was successfully introduced into *L. Lactis* C20 (Figure S8), resulting in a substantial increase in Nisin titers. Specifically, the growth status of the Nis-1 and Nis-2 strains is not significantly different from that of the wildtype strain, indicating that the introduction of pNZ8148-Nis1 or pLEB124-Nis2 will not cause growth defects in the strains (Figure 5A). However, in the Nis-12 strain, it could be seen from the growth period that the strain has a slower growth rate in the initial stage, taking about 12 h to enter the exponential growth stage. Moreover, compared with other strains, the Nisin-12 strain entered the exponential growth phase at a later time point, indicating that the introduction of the dual-plasmid system reduced the growth rate of the Nis-12 strain, thereby delaying the onset of the exponential growth period (Figure 5A).

Starting at the eighth hour, the Nisin titer of the Nis-1 strain was significantly higher than that of the C20 strain, indicating that the overexpression of the Nis-1 gene cluster could substantially enhance the Nisin titer (Figure 5B). In addition, the Nisin titers of Nis-2 and Nis-12 strains were consistently higher than those of Nis-1 strain throughout the fermentation period. This suggested that the overexpression of the Nis-2 and Nis-12 gene clusters significantly exceeded that of Nis-1 gene cluster in enhancing Nisin titers. In addition, at 32 h, the Nisin titers reached their peak in the C20 and Nis-1 strains, registering 2381.56 IU/mL and 2915.62 IU/mL, respectively, which represented a significant increase in Nisin titers of 22.4% compared to the original C20 strain (Figure 5B). Notably, there was a sudden increase in Nisin titer of *L. lactis* Nis-2 and Nis-12 at 12 h with a peak value of 3945.42 IU/mL and 3883.79 IU/mL at 40 h, respectively, leading to a 2.21-fold and 2.17-fold increase in Nisin titer compared with the original strains (Figure 5B). This suggests that when introducing the Nis-2 gene cluster and the Nis-12 gene cluster, the Nisin titers of the Nis-2 and Nis-12 strains significantly increased compared to the C20 and Nis-1 strains. However, there was no significant difference in Nisin titers between Nis-2 and Nis-12, indicating that overexpression of the Nis-2 gene cluster and dual-plasmid system overexpression of the Nis-12 gene cluster have a comparable effect on promoting Nisin biosynthesis, which proved that introducing certain regions of the Nisin biosynthetic gene cluster using PMS strategy was feasible for improving the Nisin titer in *L. lactis*.

### 2.5. Expression Profiles of Genes Within Nisin Biosynthetic Gene Clusters in Engineered L. lactis Strains

**Figure 6:**
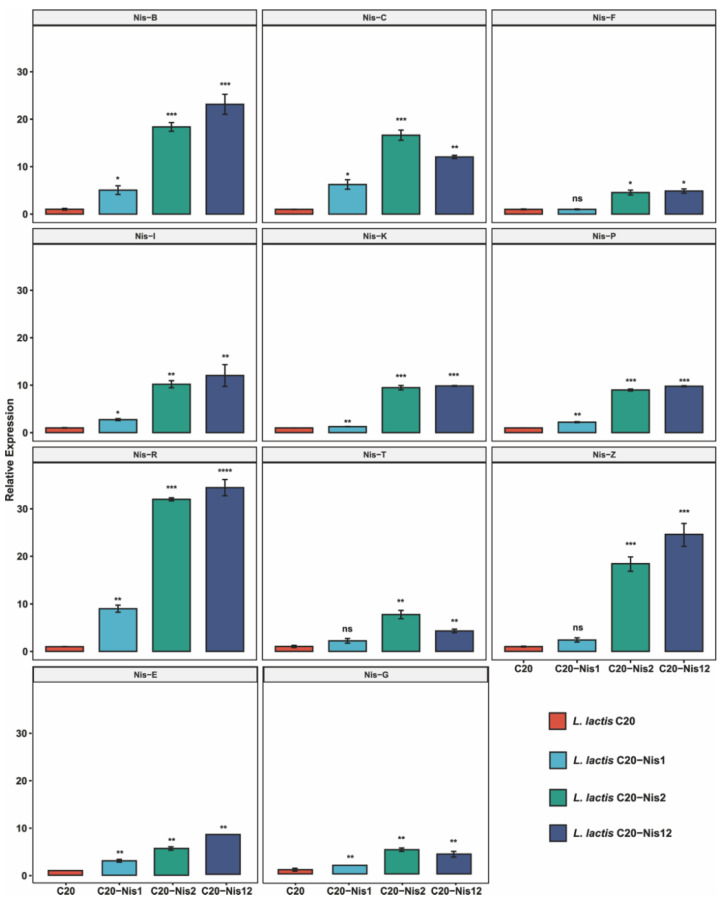
Relative expression levels of genes within Nisin biosynthetic gene cluster in *L. lactis* C20 and engineered *L. lactis* Nis-1, *L. lactis* Nis-2, and *L. lactis* Nis-12 by quantitative Real-Time PCR (qRT-PCR) analysis. Nis-Z, the precursor of Nisin; Nis-B, Nisin biosynthesis protein; Nis-T, ABC transporter ATP-binding protein; Nis-C, Nisin biosynthesis protein; Nis-I, lantibiotic immunity lipoprotein; Nis-P, serine protease; Nis-R, DNA-binding response regulator; Nis-K, sensor histidine kinase; Nis-F, ABC transporter ATP-binding protein; Nis-E, lantibiotic ABC transporter permease, Nis-G, transporter. Expression is presented relative to that of the control genes of *L. lactis* C20. Data are presented as means ± SD from three parallel replicates. ns, no significance; *, *p* < 0.05; **, *p* < 0.01; ***, *p* < 0.001; ****, *p* < 0.0001.

There are significant differences in the expression levels among the different engineered *L. lactis* strains, reflecting distinct regulatory impacts of the nisin-producing strains (Figure 6). It is worth noting that genes Nis-B and Nis-E show significant upregulation in *L. lactis* Nis2 and Nis12, suggesting that these variants strongly induce certain genes within the gene cluster. The gene Nis-C exhibits the highest expression in *L. lactis* Nis12, implying a synergistic effect when both *L. lactis* Nis1 and Nis2 are present. Moreover, genes Nis-T, Nis-Z, and Nis-R demonstrate significant upregulation in both individual and combined engineered *L. lactis* strains, highlighting their sensitivity to the Nisin-based regulation. On the contrary, genes Nis-F, Nis-K, and Nis-P show no significant change or slight downregulation during the treatments, indicating possible inhibition or lack of response to these specific conditions. The gene Nis-R shows the most pronounced response under the Nis2 conditions, with over a 20-fold increase compared to the control group (Figure 6). This dramatic upregulation hints at a possible regulatory node within the biosynthesis pathway that is particularly sensitive to the presence of *L. lactis* Nis-2. The genes Nis-T and Nis-Z are significantly upregulated in multiple engineered strains and may play a critical role in the early stages of Nisin biosynthesis or transport, as their expression levels were consistently responsive to the presence of external Nisin. The gene Nis-G, despite showing minimal changes across different conditions, maintains a basal level of expression critical for the stability and functionality of the Nisin biosynthesis cluster, possibly serving a housekeeping role within the Nisin pathway. The gene expression correlations observed across different conditions suggest a coordinated regulation of the Nisin biosynthesis gene cluster. The expression of genes such as Nis-T, Nis-Z, and Nis-R appears to be tightly linked, potentially forming a regulatory subcluster within the larger gene set. This coordination could be crucial for the efficient synthesis and processing of Nisin, ensuring optimal production and secretion under varying environmental pressures.

### 2.6. Nisin Titers of L. lactis Nis-2 in a 5 L Bioreactor

**Figure 7:**
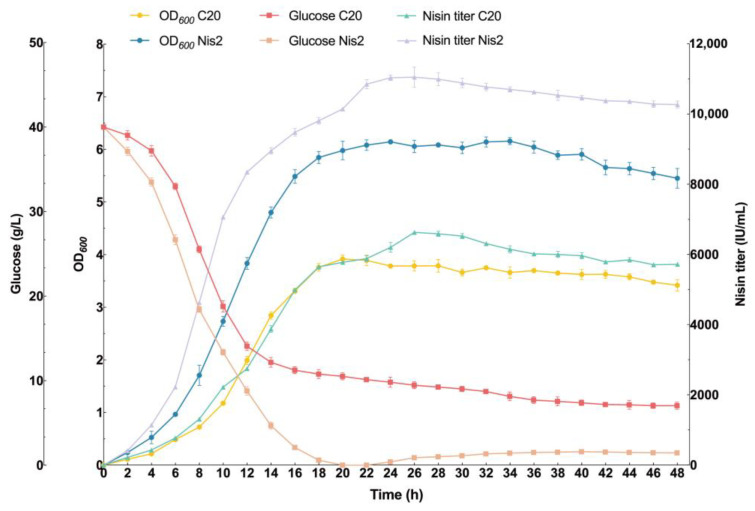
Fed-batch fermentation for Nisin production in a 5 L bioreactor by *L. lactis* C20 and engineered *L. lactis* Nis-2. Time courses of cell density, concentration of residual glucose, and Nisin titer of *L. lactis* C20 and *L. lactis* Nis-2 during the fed-batch fermentation. Glucose C20, residual glucose concentration of fermentation broth in *L. lactis* C20; Glucose Nis2, residual glucose concentration of fermentation broth in *L. lactis* Nis-2; OD_600_ C20, cell density of *L. lactis* C20 during fermentation; OD_600_ Nis2, cell density of *L. lactis* Nis-2 during fermentation; Nisin titer C20, Nisin titer of *L. lactis* C20 during fermentation; Nisin titer Nis2, Nisin titer of *L. lactis* Nis-2 during fermentation. Data are presented as means ± SD from three parallel replicates.

In this study, we monitored the fermentation kinetics of two *L. lactis* strains, designated as *L. lactis* C20 and engineered *L. lactis* Nis2, using a 5 L fermenter for the production of Nisin. The evaluated parameters include optical density at 600 nm (OD_600_), residual glucose concentration, and Nisin titer within 48 h. The OD_600_ of both strains will initially increase, and *L. lactis* Nis2 has a faster growth rate compared to *L. lactis* C20. The OD_600_ of *L. lactis* Nis2 reached a peak of about 6.2 at 34 h, whereas *L. lactis* C20 peaked at around 3.8 at 20 h (Figure 7). After reaching their respective peaks, the OD_600_ of both strains slightly decreased, and *L. lactis* Nis2 maintained a higher OD_600_ throughout the fermentation process compared to *L. lactis* C20. Both strains demonstrated a rapid consumption of glucose during the initial phase of fermentation. The residual glucose concentration dropped sharply in the first 12 h, with *L. lactis* Nis2 depleting the glucose more rapidly than *L. lactis* C20. By 18 h, the glucose concentration of *L. lactis* Nis2 was close to zero, indicating complete substrate utilization. With the start of the feeding process, the glucose concentration of *L. lactis* Nis2 slightly increased, allowing the strains to utilize glucose for growth and metabolism, which was reflected in the continued increase in biomass and Nisin titers. Nisin production was tracked over the fermentation. Clearly, *L. lactis* Nis2 produced higher Nisin titers compared to *L. lactis* C20, reaching a peak of 11,052.9 IU/mL at 26 h (Figure 7), which is currently reported as the highest Nisin titer produced in microbial cells through systematically genetic regulation of a Nisin biosynthetic gene cluster. In contrast, *L. lactis* C20 reached a maximum Nisin titer of 6629.1 IU/mL at 26 h (Figure 7). After the peak production, both strains displayed a slight decrease in Nisin titers; however, *L. lactis* Nis2 still maintained a higher Nisin titer throughout the fermentation compared to *L. lactis* C20. Accordingly, the final Nisin titer of *L. lactis* Nis2 was 2.8-fold higher than that of the shake flask.

## 3. Discussion

In this study, we proposed and designed a tool to break through the barrier of the RM system based on the pre-modification system, thereby significantly improving the transformation efficiency in *L. lactis* for the first time. Accordingly, the RM systems along with their respective recognition motifs were identified, and PMS tools were constructed for pre-modifying plasmids before transformation into *L. lactis*, achieving high transformation efficiency in genetically intractable *L. lactis* strains. Compared with current methods, such as the RM-silent “SyngenicDNA” strategy [18] and bypassing plasmid DNA through the restriction-negative, modification-proficient nature strains [29], our study offers a more rapid and efficient tool for genetic manipulation in *L. lactis*. The significant improvement of transformation efficiency in *L. lactis* C20 is a crucial step in making LAB more genetically accessible, thereby deepening our understanding of the genetic modification and metabolic engineering of *L. lactis*.

According to reports, the use of plasmids that have not been methylated by the RM systems of the target strains can improve the transformation efficiency [11,32,33]. This may be due to the presence of highly active type IV RM systems in some strains, which recognize and cleave the methylated plasmids. However, such systems are absent in *Lactococcus lactis* C20, and the unmethylated plasmids of the RM systems show lower transformation efficiency. Therefore, the PMS strategy was adopted to circumvent the barriers of the RM system. In addition, our findings are consistent with recent studies that indicate a method for overcoming the barrier of certain type I RM genes to gene manipulation in specific strains, such as *Streptococcus* spp., *Staphylococcus* spp., and *E. coli* [29,34,35,36,37,38,39,40]. Compared to methods involving random or partial expression of RM-related genes, our strategy is more specific and efficient, achieving higher efficiency at lowest cost.

Although PMS tools have achieved significant improvements in transformation efficiency, it cannot be ruled out that there are some less active and non-identified RM systems that may hinder further improvements in transformation efficiency. As a solution, we designed the PMS-RCA tool to further improve transformation efficiency. The recent literature reports that RCA products of plasmids can spontaneously circulate to form complete plasmids within *E. coli* [31]. Based on this, we hypothesize that the unique characteristics of concatemeric RCA products may overcome certain limitations of the RM systems through altering the spatial conformation of DNA recognized by the RM systems and reducing the frequency of cleavage (Figure S4). Thus, the PMS-RCA tool aims to generate a mix product that could further break through the limitation of RM systems, thereby further improving transformation efficiency. In addition, another advantage of RCA is the generation of a large amount of transformed DNA due to the amplification process. This is particularly beneficial when working with low-copy plasmids or challenging host organisms with low transformation efficiencies. The amplified DNA produced by RCA provides more transformed DNA molecules available for uptake by recipient cells, thereby increasing the chances of successful transformation events [41]. Moreover, RCA can perform multiple rounds of replication, resulting in the production of a high copy number of transformed DNA within the recipient cells. This feature is particularly advantageous when aiming to achieve high-level gene expression or when the introduced DNA needs to be integrated into the host genome. The increased copy number enhances the expression levels of transgenes and facilitates their stable integration, resulting in improved transformation efficiency [42]. By using PMS-RCA tools, the transformation efficiency further substantially increases, which is a promising strategy to evade the barrier caused by the RM systems in *L. lactis*.

In order to further enhance the Nisin biosynthetic capacity of *L. lactis* C20, we successfully overexpressed the Nis-1, Nis-2, and Nis-12 gene clusters in the Nisin biosynthetic pathway for the first time in *L. lactis*, resulting in a significant increase in Nisin titers. The previous literature provided individual expression examples of genes in the Nisin biosynthetic gene cluster. However, these studies indicated that Nisin titers only slightly increased, with an increase of no more than 50% [43,44,45]. Hence, we divided the full-length Nisin biosynthetic gene cluster into two parts: Nis-1 gene cluster (including *nisZBTCIP*), primarily responsible for the supply of Nisin precursors, and Nis-2 (including *nisRKFEG*), primarily involved in the regulation and immunity of the Nisin synthesis [46]. We evaluated the growth status and Nisin titers of the engineered strains *L. lactis* Nis-1, *L. lactis* Nis-2, and *L. lactis* Nis-12 overexpressing the Nis-1, Nis-2, and the Nis-12 gene clusters, respectively. The variability in gene expression reflects biochemistry mechanism for Nisin biosynthesis and regulation. By modulating the expression of the biosynthesis genes, Nisin-producing bacteria can adjust their production of this potent antimicrobial peptide to better compete with neighboring microbes in a diverse microbial ecosystem [47]. This regulation not only maximizes the effectiveness of Nisin under different ecological conditions but also minimizes metabolic costs associated with its overproduction [48]. The differential expression of the Nisin biosynthesis gene cluster under the influence of different *L. lactis* strains provides valuable insights into the genetic control mechanisms that regulate Nisin production. These findings highlight the complexity of gene regulation in the secondary metabolite pathways and underscore the adaptability of microbial producers in response to biological factors, such as the presence of antimicrobial peptides. Further studies focusing on the interaction between these regulatory patterns and other cellular pathways will deepen our understanding of microbial competitiveness and the evolutionary dynamics of antibiotic production. These results not only underscore the impact of different *L. lactis* strains on gene expression but also suggest potential feedback mechanisms inherent to the Nisin biosynthesis pathway. The observed differential expression patterns suggest that the regulatory network controlling Nisin biosynthesis is complex, and certain genes are more responsive to external Nisin concentrations [49]. This regulation may reflect an adaptive biochemistry mechanism through which Nisin-producing bacteria can finely tune their antimicrobial peptide production in response to the environmental cues [50]. Therefore, this multi-copy expression strategy significantly enhanced the Nisin titer, particularly with the *L. lactis* Nis2 and *L. lactis* Nis12 strains exhibiting higher activity compared to the *L. lactis* Nis1 strain, underscoring the critical role of the immune regulatory segment (Nis2) in Nisin biosynthesis. Through fermentation experiments and gene expression analyses, this study further elucidated the complex regulatory mechanisms governing Nisin synthesis, suggesting the presence of potential feedback and adaptive regulatory mechanisms. This highlights the substantial industrial production potential of the strain and provides an effective metabolic engineering strategy for enhancing Nisin biosynthesis.

The fermentation profiles of *L. lactis* C20 and Nis2 in a 5 L bioreactor reveal significant differences in their growth kinetics, glucose consumption, and Nisin biosynthesis capabilities. The faster growth rate observed in *L. lactis* Nis2 suggests that this strain may possess a higher metabolic activity or better adaptation to the fermentation conditions compared to *L. lactis* C20. The sustained higher OD_600_ of *L. lactis* Nis2 throughout the fermentation period indicates more robust cell growth, which is crucial for high-yield Nisin production [51]. Both strains efficiently utilized the available glucose, with a rapid decline observed within the first 12 h. The faster glucose depletion by *L. lactis* Nis2 correlates with its higher growth rate and metabolic activity. During the initial 12 h of fermentation, the glucose consumption rate significantly accelerated, resulting in a notable increase in biomass growth rate and the Nisin titers. This indicates that glucose is a significant factor influencing biomass growth and metabolism at this stage. Relatively sufficient glucose is beneficial for the biosynthesis of Nisin in the strains. *L. lactis* Nis2 exhibits excellent capacity for Nisin biosynthesis, with a peak titer more than double that of strain C20. This higher productivity could be attributed to genetic or regulatory differences between strains, enhancing the Nisin biosynthesis pathways in the strain Nis2. The slight decrease in Nisin titers after the peak production phase could be due to the depletion of glucose or feedback inhibition mechanisms [52]. However, *L. lactis* Nis2 maintained a higher residual Nisin titer, suggesting better stability or lower degradation rates compared to *L. lactis* C20, highlighting the potential of the strain Nis2 for the industrial Nisin production due to its higher growth rate, efficient glucose utilization, and excellent Nisin yield. Further investigations on the genetic and metabolic pathways of *L. lactis* Nis2 could provide insights for optimizing fermentation conditions and expanding production. In addition, exploring the stability and degradation mechanisms of Nisin during fermentation could help maintain higher yields and improve overall process efficiency [53].

## 4. Materials and Methods

### 4.1. Bacterial Strains, Plasmids, Culture Media, and Conditions

All bacterial strains/plasmids used in this study are summarized in Table S1. *E. coli* W3110 and *E. coli* MC1601 were grown aerobically in Luria–Bertani (LB) medium at 37 °C (1% tryptone, 0.5% yeast extract, and 1% NaCl) and used as cloning hosts. *L. lactis* C20 was incubated statically in seed medium at 30 °C (1.5% yeast extract, 1.5% peptone, 2% sucrose, 0.15% NaCl, 2% KH_2_PO_4_, and 0.015% MgSO_4_∙7H_2_O) for culture and fermentation medium (1.5% yeast extract, 1.5% peptone, 2% glucose, 0.15% NaCl, 2% KH_2_PO_4_, 0.5% corn steep liquor, 0.3% cysteine, and 0.015% MgSO_4_∙7H_2_O) for fermentation. Appropriate antibiotics were supplemented as necessary: 180 and 5 μg/mL erythromycin for *E. coli* and *L. lactis*, respectively; 50 and 5 μg/mL chloramphenicol for *E. coli* and *L. lactis*, respectively; 100 μg/mL ampicillin for *E. coli*; 100 μg/mL spectinomycin for *E. coli*.

### 4.2. SMRT Sequencing and Methylome Analysis

Genomic DNA was extracted from 5 mL of overnight cultures of *L. lactis* C20 cells using a SPARKeasy Bacteria DNA Kit (SparkJade, Qingdao, China) following the manufacturer’s protocol. The *L. lactis* C20 genome was sequenced using the PacBio RS II platform and Illumina HiSeq 4000 platform at the Beijing Genomics Institute (BGI, Shenzhen, China). The principle of single-molecule, real-time sequencing (SMRTseq) and related base modification detection has been detailed previously [54]. To identify the modified positions, Pacific Biosciences’ SMRTanalysis (PacBio, Menlo Park, CA, USA) was used to detect the modified base positions.

### 4.3. Classification of RM System

RM system identification was performed using the SEQWARE computer resource and a BLAST-based software (v2.16.0) module combined with the curated restriction enzyme database (REBASE), as described earlier [17,55]. To identify the MTase target sequence motifs, the first 1000 kinetic hits were selected, and a ±20 base window around the detected base was subjected to MEME-ChIP [56]. Another round of MEME-ChIP analysis was performed on this 1% population of sequence context to confirm the absence of any additional consensus motifs.

### 4.4. Construction of PMS System

Selected MTase genes from *L. lactis* C20 genome and homologous arms from *E. coli* W3110 genomic DNA were amplified by PCR. These fragments were assembled via seamless cloning to create editing templates, which were gel-purified prior to electroporation. The pRedCas9 plasmid was electrotransformed into *E. coli* W3110 as described previously [57]. The first round of genome editing involved transforming the cells with the 100 ng editing template and the 100 ng gRNA-Dcm plasmid containing a gRNA that targeted the *dcm* gene. The edited colonies were identified using antibiotic resistance selection, colony PCR, and DNA sequencing. Subsequently, the plasmids were cured, resulting in a plasmid-free strain of *E. coli* PMS1 (carrying MTase1 of *L. lactis* C20). Unless specified otherwise, each electroporation reaction included the 100 ng editing template (dsDNA) and 100 ng gRNA plasmid. In the second round, the gRNA-HsdM plasmid and the editing template were electroporated into the cells, resulting in the production of the *E. coli* PMS2 (carrying MTase1 and MTase2 of *L. lactis* C20). The last round targeted the *dam* gene with the gRNA-Dam plasmid and the editing template, resulting in the generation of the *E. coli* PMS3 strain (carrying complete methylation profile of *L. lactis* C20). Finally, the plasmids were cured, leading to the development of the completely engineered *E. coli* PMS3 strain. For *E.coli* strains, electrocompetent cells were prepared as previously described [58]. The detailed workflows of constructing genome editing plasmids and gRNA plasmids are illustrated in Figure S1. All primers used in this work are shown in Table S2.

### 4.5. Overexpression of Nisin Biosynthesis Gene Cluster and Nisin Titer Assessment

To construct Nisin biosynthesis gene cluster expression plasmids, the gene cluster was divided into two segments. Each segment was then assembled onto the pLEB124 and pNZ8148 expression plasmids. A 7.0 kb DNA fragment containing half of the Nisin biosynthesis gene cluster and a 3.0 kb vector fragment from pNZ8148 were amplified, purified, and seamlessly cloned to create the pNZ8148-Nis1 plasmid. A similar method was employed for constructing the pLEB124-Nis2 plasmid with the remaining gene cluster region. The Nisin yield was quantified using an adapted agar well diffusion method [59]. A mixture of 500 μL broth and an equal volume of 0.02 M HCl was subjected to heat in a boiling water bath for 5 min, followed by centrifugation at 8000 rpm for 5 min to remove the cells and dilution with 0.02 M HCL. *M. luteus* (10^7^ CFU/mL) was inoculated into LB agar supplemented with Tween 20, and the medium was poured into plates for well creation. Standard Nisin solutions and samples were loaded into wells and incubated, and the inhibition zones were measured to determine Nisin yield. The construction processes are detailed in Figure S3, and primers are listed in Table S2.

### 4.6. Rolling Circle Amplification (RCA)-Combined PMS

The RCA procedure was conducted in accordance with the manufacturer’s instructions provided with phi29 DNA Polymerase (Beyotime, Shanghai, China). Full details of preparations and protocols are provided in the Supporting Information.

### 4.7. L. lactis Transformations and Determination of Transformation Efficiency

Electrocompetent *L. lactis* cells were prepared following a modified version of the protocol by Holo and Ahrne [60,61]. Briefly, overnight cultures were diluted to an OD_600_ value of 0.2 and cultivated at 30 °C until reaching an OD_600_ value of 0.3–0.5. Cells were then chilled, harvested by centrifugation, washed with ice-cold sterile water and glycerol, and resuspended in 10% glycerol before storage at −80 °C. For electroporation, thawed competent cells were mixed with 100 ng of plasmid DNA in an electroporation cuvette, incubated on ice for 10 min, and electroporated at 2.5 kV. After electroporation, cells were recovered in seed medium, diluted, and plated on agar with the appropriate antibiotic for a 48 h incubation period.

The transformation efficiencies were calculated using data from three independent experiments. For each independent experiment, a single 100 μL aliquot of electrocompetent L. lactis cells was used for all plasmids. To quantify transformation efficiency, colony-forming units (CFU) were enumerated after plating serial dilutions on the agar plates. The transformation efficiencies for individual plasmids within experiments were determined as the average of CFU counts from three replicate agar plates, presented as CFU per μg of plasmid DNA.

### 4.8. Quantitative Real-Time PCR

*L. lactis* strains were cultured in seed medium for 30 h and subsequently diluted to achieve uniform cell density. Total RNA was extracted using an RNAprep Pure Cell/Bacteria Kit (TIANGEN BIOTECH, Beijing, China) and reverse-transcribed into first-strand cDNA with a SPARKscript II RT Plus Kit (SparkJade, Shandong, China). The gene expression levels of *nis-Z*, *nis-B*, *nis-T*, *nis-C*, *nis-I*, *nis-P*, *nis-R*, *nis-K*, *nis-F*, *nis-E*, and *nis-G* were quantified by quantitative Real-Time PCR (qRT-PCR) utilizing 2xSYBR Green qPCR Mix (SparkJade, Shandong, China). The 16s rRNA gene was used as the housekeeping gene [62] and data analysis was conducted using the comparative CT (2^−ΔΔCT^) method.

### 4.9. Flask Fermentation and Fed-Batch Fermentation

Following overnight cultivation, *L. lactis* strains were inoculated into seed medium and incubated for 8 h to prepare seed cultures. For flask fermentation experiments, 250 mL Erlenmeyer flasks containing 100 mL of seed fermentation medium were utilized. A 1 mL aliquot of the seed culture was inoculated into static flasks and incubated at 30 °C for 48 h. Samples were collected at 2 h intervals to analyze cell density, broth pH, and Nisin titer. Fed-batch fermentation was carried out in a 5 L fermenter (BSINSS, Shanghai, China) at 30 °C for 48 h under anaerobic conditions. The seed culture was inoculated into the bioreactor containing 3 L of medium with a 10% inoculum. The pH of the fermentation broth was maintained at 6.0 by the addition of 5 M NaOH throughout the fermentation process. Samples were collected every 2 h to measure cell density, residual glucose concentration, and Nisin titer.

### 4.10. Quantification and Statistical Analysis

An amperometric biosensor based on glucose oxidase was used for the quantification of glucose concentration using an SBA-90 biosensor (Biology Institute of Shandong Academy of Sciences, China). Optical density (OD) values were measured using a Varioskan™ LUX Multimode Microplate Reader (Thermo Fisher Scientific Inc., Waltham, MA, USA) at a wavelength of 600 nanometers. All experimental data were statistically analyzed using Student’s *t*-test, and *p* < 0.05 was considered statistically significant.

## 5. Conclusions

Taken together, our studies have developed RM-silent PMS tools that systematically evade the RM barrier for enhancing Nisin biosynthesis through efficient genetic engineering in *L. lactis*. This strategy not only enhances the capabilities for in-depth genetic analysis and metabolic engineering of *L. lactis* but also has the potential to be applied to other genetic toolkits where low transformation efficiency constrains genetic manipulation in limited *L. lactis*.

## Data Availability

Data is contained within the article or Supplementary Materials.

## Supplementary Materials

The following Supporting Information can be downloaded at: https://www.mdpi.com/article/10.3390/ijms26052200/s1. Reference [63] is cited in the supplementary materials.
