# Supplementary material for: Development of a Pre-Modification Strategy to Overcome Restriction–Modification Barriers and Enhance Genetic Engineering in Lactococcus lactis for Nisin Biosynthesis
Source: Int J Mol Sci. 2025 Feb 28;26(5):2200. doi: 10.3390/ijms26052200 (PMC11900431; doi:10.3390/ijms26052200)
Supplement: Supplementary file 1 [file ijms-26-02200-s001.zip › ijms-3504514-supplementary.pdf]

Development of a Pre-Modification Strategy to Overcome Restriction–Modification Barriers and Enhance Genetic Engineering in *Lactococcus lactis* for Nisin Biosynthesis

Chen Chen [1,2,3], Yue Zhang [1,2,3], Ruiqi Chen [1,2,3], Ke Liu [3], Hao Wu [3,*], Jianjun Qiao [1,2,3] and Qinggele Caiyin [1,2,3,*]

1 School of Chemical Engineering and Technology, Tianjin University, Tianjin 300072, China; cc_2019@tju.edu.cn (C.C.); 16602659785@163.com (Y.Z.); chenruiqi0803@163.com (R.C.); jianjunq@tju.edu.cn (J.Q.)

2 State Key Laboratory of Synthetic Biology, Tianjin University, Tianjin 300072, China

3 Zhejiang Institute of Tianjin University, Shaoxing 312300, China; hllk@163.com

* Correspondence: dream72wh@tju.edu.cn (H.W.); qinggele@tju.edu.cn (Q.C.); Tel./Fax: +86-22-8740-2107 (Q.C.)

Detailed protocol for RCA

1.Reaction system

Reagent	Volume	Final Concentration
Nuclease-free Water	39 μl	-
10X Reaction Buffer	5 μl	1X
dNTP (2.5 mM each)	2 μl	100 μM
Random Hexamer Primers (100μM)	2 μl	4 μM
Template DNA (≥1 ng)	1 μl	5 ng/μl
Total Volume	49 μl	-

2. Pre-denaturation of Template DNA: Place the above reaction system in a PCR machine and incubate at 95°C for 5 minutes, then quickly transfer it to an ice bath for 2 minutes or more.

3. Isothermal amplification reaction: Add 1 μl phi29 DNA Polymerase to the cooled reaction system, incubate at 30°C for 2-16 hours. It is usually sufficient to incubate for only 2 hours. If more amplification product is desired, the incubation time can be extended to 16 hours. It is recommended to use a thermal bath for the reaction. If using a PCR machine for the reaction, adjust the temperature of the lid to 40°C to avoid enzyme inactivation.

4. Termination reaction: Incubate at 65°C for 10 minutes.

5. Detection of amplified product: The amplified product can be detected by various methods including gel electrophoresis, hybridization, and fluorescence-based assays.

Table S1. All strains and plasmids used in this study.

Strains/Plasmids	Description	Source
**Strains**		
E.coli W3110	Cloning host for construction of Pre-Modification System (PMS)	Laboratory stock
L.lactis F44	Non-model strain belonging to *L. lactis* subsp. *cremoris*, low yield of Nisin producer with normal transformation of foreign genetic elements	Laboratory stock
L.lactis C20	Non-model strain belonging to *L. lactis* subsp. *cremoris*, high yield of Nisin producer with lower transformation of foreign genetic elements	Laboratory stock
E.coli W3110 /pRedCas9	*E.coli* W3110 derivative harboring plasmid pRedCas9	This study
E.coli W3110 /pRedCas9-pGRB1	*E.coli* W3110/pRedCas9 derivative harboring plasmid pGRB for PMS1	This study
E.coli W3110 /pRedCas9-pGRB2	*E.coli* W3110/pRedCas9 derivative harboring plasmid pGRB for PMS2	This study
E.coli W3110 /pRedCas9-pGRB3	*E.coli* W3110/pRedCas9 derivative harboring plasmid pGRB for PMS3	This study
E.coli PMS1	*E.coli* W3110 derivative with MTase1 integrated in the gnenome	This study
E.coli PMS2	*E.coli* PMS1 derivative with MTase1 and MTase2 integrated in the gnenome	This study
E.coli PMS3	*E.coli* PMS2 derivative with MTase1, MTase2 and MTase3 integrated in the gnenome	This study
L.lactis C20 /pNZ8148	*L.lactis* C20 derivative harboring plasmid pNZ8148	This study
L.lactis C20 /pLEB124	*L.lactis* C20 derivative harboring plasmid pLEB124	This study
L.lactis C20 /pNZTS-Cas9	*L.lactis* C20 derivative harboring plasmid pNZTS-Cas9	This study
L.lactis C20 /pNZTS-cBE	*L.lactis* C20 derivative harboring plasmid pNZTS-cBE	This study
L.lactis Nis-1	*L.lactis* C20 derivative harboring plasmid pNZ8148-Nis1	This study
L.lactis Nis-2	*L.lactis* C20 derivative harboring plasmid pLEB124-Nis2	This study
L.lactis Nis-12	*L.lactis* C20 derivative harboring plasmid pNZ8148-Nis1 and pLEB124-Nis2	This study

**Plasmids**		
pNZTS-Cre	Amplification of repA(Temperature Sensitive)-Ery region	Laboratory stock
pLEB124	*L. lactis* secretion vector harboring P_{45} promoter	Laboratory stock
pNZ8148	*L. lactis* NICE vector harboring P_{nisA} promoter	Laboratory stock
pNZTS-cBE	dCas9-based cytosine base editors for *L.lactis*	[1]
pNZTS-Cas9	Cas9-based genome editing system for *L.lactis*	This study
pRedCas9	SpCas9-based genome editing system for *E.coli*	[2]
pGRB	Construction of sgRNA expression cassette	[2]
pGRB-1	pGRB derivative with sgRNA for PMS1	This study
pGRB-2	pGRB derivative with sgRNA for PMS2	This study
pGRB-3	pGRB derivative with sgRNA for PMS3	This study

Table S2. All primers used in this study.

Name	Sequence (5'-3')
BsaI-MTase1-arm-up-F	GGCTACGGTCTCCGATGGCCTCGCCATAGTAGCGATCGAACA
inFus-MTase1-arm-down-F	GACTGGGCCTTTCGTTTTATCTGTTGTTTGTCGGTGAACGCTCTCGGCTGACTAACTAATTACACCTTCTC
MTase1-Ter-R	ATAAAACGAAAGGCCCAGTCTTTCGACTGAGCCTTTCGTTTTATTTGATGCCTGGCTATATTGAGAAAGCCTCTAATAAG
MTase1-arm-down-inFus-R	AGGACTGAGCTAGCCGTCAATTCTCAAGGAGAAGCGGATGAAAC
MTase1-arm-down-sgRNA	AGGACTGAGCTAGCTGTCAAAGCGATCAAACCGCGTCTGG
P-MTase1-F	TTGACGGCTAGCTCAGTCCTAGGTACAGTGCTAGCTACTAGTGAAAGAGGAGAAATACTAGATGCTAAACCATAACGGTAT
sgRNA1	TTGACAGCTAGCTCAGTCCTAGGTATAATGCTAGCATACGATGTTAACGCGTGAGGT
BsaI-MTase2-arm-up-F	ATCTAAGGTCTCCTGCGACGGCGCTCCCTTCCCTTTCA
MTase2-arm-down-BsaI-R	AGGACTGAGCTAGCTGTCAATGATATCACTGGCAGGCACAGTTCGA
MTase2-arm-down-F	GACTGGGCCTTTCGTTTTATCTGTTGTTTGTCGGTGAACGCTCTCGAGATTTCACCGGCCATCTGA
MTase2-arm-down-inFus-R	AGGACTGAGCTAGCCGTCAAGGCCACTCGCAGCAAAAATATG
MTase2-Ter-R	ATAAAACGAAAGGCCCAGTCTTTCGACTGAGCCTTTCGTTTTATTTGATGCCTGGCTATCGATTTTCAAGAAGGGCGATC
P-MTase2-F	TTGACGGCTAGCTCAGTCCTAGGTACAGTGCTAGCTACTAGTGAAAGAGGAGAAATACTAGATGAACCCACCATACTCACTC
sgRNA2	TTGACAGCTAGCTCAGTCCTAGGTATAATGCTAGCCCGCCTGTTTGATTTTTGGCGT
BsaI-MTase3-arm-up-F	GGCTACGGTCTCCACTGATTGGATGACCAACACCACTTTC
MTase-arm-down-R	AGGACTGAGCTAGCCGTCAATTATATGTGCGGGGAAATTGCCGGAG
MTase3-arm-down-F	GACTGGGCCTTTCGTTTTATCTGTTGTTTGTCGGTGAACGCTCTCGTGTGAGTTCCGTAAATTAAGCAGCG
MTase3-arm-down--R	AGGACTGAGCTAGCTGTCAACGATGAAGTGGTGAGTGTAAAATCG
MTase3-rbs31-R	ATTTCTCCTCTTTCTCTAGATTAAACTAGTGCTTCTTCTT
MTase31-F	TCTAGAGAAAGAGGAGAAATACTAGATGATGATTGTTCCGCTCGCCGAT
MTase31-R	ATAAAACGAAAGGCCCAGTCTTTCGACTGAGCCTTTCGTTTTATTTGATGCCTGGCTATAATTCGTTAGAAACTGC

P-MTase3-F	TTGACGGCTAGCTCAGTCCTAGGTACAGTGCTAGCTACTAGTGAAAGAGGAGAAATACTAGATGTTTGTTCAATCTATGAAGTTC
sgRNA3	TTGACAGCTAGCTCAGTCCTAGGTATAATGCTAGCCACATCATCGGTACAGTTCTGT
pGRB-F	ACTCGTGCACCCAACTGATCTTCA
pGRB-HindIII-R	GGCTACGGTCTCCCATCGTGGCACTGGCCGTCGTTTTACAA
pGRB-R	AGTCCTGTCGGGTTTCGCCA
pGRB-XbaI-F	GGCTACGGTCTCCGCATAAGGATCCCCGGGTACCGAGCTCGAA
ad3-F	GACTGGGCCTTTCGTTTTATCTGTTGTTTGTCGGTGAACGCTCTCGAGGATGAAGTGTATACGTGTCAGGC
ad3-R	AGGACTGAGCTAGCTGTCAAATAGATAAACAGGTGCGTCGTCTGG
sg1-4	GGCTACGGTCTCCATGCGGGTGCCACTTTTTCAAGTTGATAACGGACTAGCCTTAT
MT3-arm-up-F	GGCTACGGTCTCCACTGAACGTACAGAACTAATGCGTAGTA
MT3-arm-up-R	AGGACTGAGCTAGCCGTCAATTATATAGTATCTACGGTCACAACTCTAATC
MT1-ins-F	GGCTACGGTCTCCGATGGCCTC
MT1-ins-R	GGCTACGGTCTCCATGCGGGTG
GpRed-F	GGCTTTATGACGTAACATCCGTTTGG
GpRed-R	CCTCTGTTCGTCTCGACATCCG
pNZTs-Cas9-F	AGAAGCAAACTTAAGAGTGTGTTGATAGT
pNZTs-Cas9-R	ACGTTAAGGGATTTTGGTCATGAGATTATCA
pNZTs-Rep-F	ATGCTCGCGTTATCGACAATAATATTATTACCA
pNZTs-Rep-R	CAATTGTTTTATTATTTGGTTGAGTACTTTTTCACTCG
DouBsaI-R	ACTCTTATCCATCAATCCATCACTGGT
DouBsaI-F	AGAAGCAAACTTAAGAGTGTGTTGATAGT
Linear-DouBsaI-F	TGACACGGTCTCGCATCTTGG
Linear-DouBsaI-R	CAATTCGGTCTCGTGCGTAATATGC
sgRNA1-1R	GGCTACGGTCTCCGACTGATCCCCCAAGATGCTAATT
sgRNA1-2F	GGCTACGGTCTCCAGTCGCAATTAAAGTTGAAAAACCCTACC
sgRNA2-2R	GGCTACGGTCTCCGTACGATCCCCCAAGATGCTAATT
sgRNA2-3F	GGCTACGGTCTCCGTACGCAATTAAAGTTGAAAAACCCTACC
pinsCas9-F	ATCGATATGCTTAGTCACCTCCTAGCTGACTCAAAT
pinsCas9-R	GGCACTCACCATGGATAAGAAATACTCAATAGGCT
pVector-F	TCTTATCCATGGTGAGTGCCTCC
pVector-R	AGGTGACTAAGCATATCGATACCG
GgRNA-F	GAGGATCCCCCAAGATGC

GgRNA-R	GATTTAAGAGTGCATATTACGCAC
GNZts-F	AGGTATACTACTGACAGCTTCC
GNZts-R	CCGCCATACCACAGATGTTC
Lac-F	CCAGTGAGCGCGCGTAATAC
Lac-R	GTATTACGCGCGCTCACTGG
Lin-DouBasI-F	CAATTCGGTCTCGTGCGTAATATGCACTCTTAAATCAAAAAAAAGAAACTG
Lin-DouBsaI-R	TGACACGGTCTCGCATCTTGGGGGATCCTCTAGAGTCTAG
Lin124-F	TATTCTAGGTTCCCTTCAGTAATACGT
Lin124-R	CATTCGAATTCCTGCAGAATTTCG
HindIII-R	GAAAAAAATTAACTTAAGTAATGGTGAGTAAAGGAGAGGAAGATAATATGGC
NcoI-F	TCTGCAGGAATTCGAATGGCTTACTTGTAAAGTTCATCCATCCCTCCTGT
s124-R	TCGCCAAAAACTGGAATATGATTC
s124-F	TTCATTCTGCTAACCAGTAAGGC
g124-F	GATGAAACGAGAGAGGATGCTCAC
g124-R	CACATCAATGCGATGTCCCAG
gNZ8148-F	GTCAGATAGGCCTAATGACTGGC
gNZ8148-R	ACGCCTGTTTTAACGATTATGCC
sgRNA1	GGCTACGGTCTCCGATGGCAATTAAAGTTGAAAAACCCTACC
sgRNA2	GCAATTAAAGTTGAAAAACCCTACCTTTACTTGCACTAATAGGTTTTATTTTATATAATTATTGAT
sgRNA4	GTTTTAGAGCTAGAAATAGCAAGTTAAAATAAGGCTAGTCCGTTATCAACTTGAAAAAGTGGCACCGAGTCGGTGCTTTTTTTCGATCGC
sgRNA5	GATCCCCCAAGATGCTAATTTATAAATAAAAAAACCACCTCTAAAAGGTGATTTTTATTTATAAATTACAGCGATCGAAAAAAAGCACCG
sgRNA6	GGCTACGGTCTCCCGCAGATCCCCCAAGATGCTAATT
dCas9-Em-F	TTCTATGAGTCGCTTTTGTAAATTTGGAAAG
dCas9-Em-R	AAAACGTCTCAAAATCGTTTCTGAGACG
Random Hexamer Primers	TCATA*A

* The 3' ends of the primers were modified with phosphorothioate.

Table S3. Methylation profiles as identified in *Lactococcus lactis* C20

No.	RM Type	Motif[a]	CP[b]	MT[c]	NMD[d]	NMG[e]	PMD[f]	MM QV[g]	MMC[h]	Partner Motif
A	II	GCGG**A**	5	^{m6}A	1544	1549	99.68	243.06	186.18	
B1	I	C**A**YNNN NNN*T*CG	2	^{m6}A	787	787	100	248.57	182.57	CGANNN-NNNRTG
B2	I	CG**A**NNN NNNR*T*G	3	^{m6}A	787	787	100	239.13	185.26	CAYNNN-NNNTCG
C	I	GCGGA**A** NDVNB	6	^{m6}A	47	236	19.92	80.47	191.12	

a The modified motif base is presented in bold, while the modified base in the complementary strand is shown in italics.
b CP, Center Position.
c MT, Modification Type
d NMD, Number of Motifs Detected
e NMG, Number of Motifs in Genome. The overall count comprises motifs present on both the "+" and "-" strands.
f PMD, Percentage of Motifs Detected.
g MMQV, Mean Modification Quality Value. The mean modification Quality Value (QV) is described as the quality score obtained from the base calls found in the motif.
h MMC, Mean Motifs Coveraged. Mean motif coverage is defined as the average depth of read coverage within a motif.

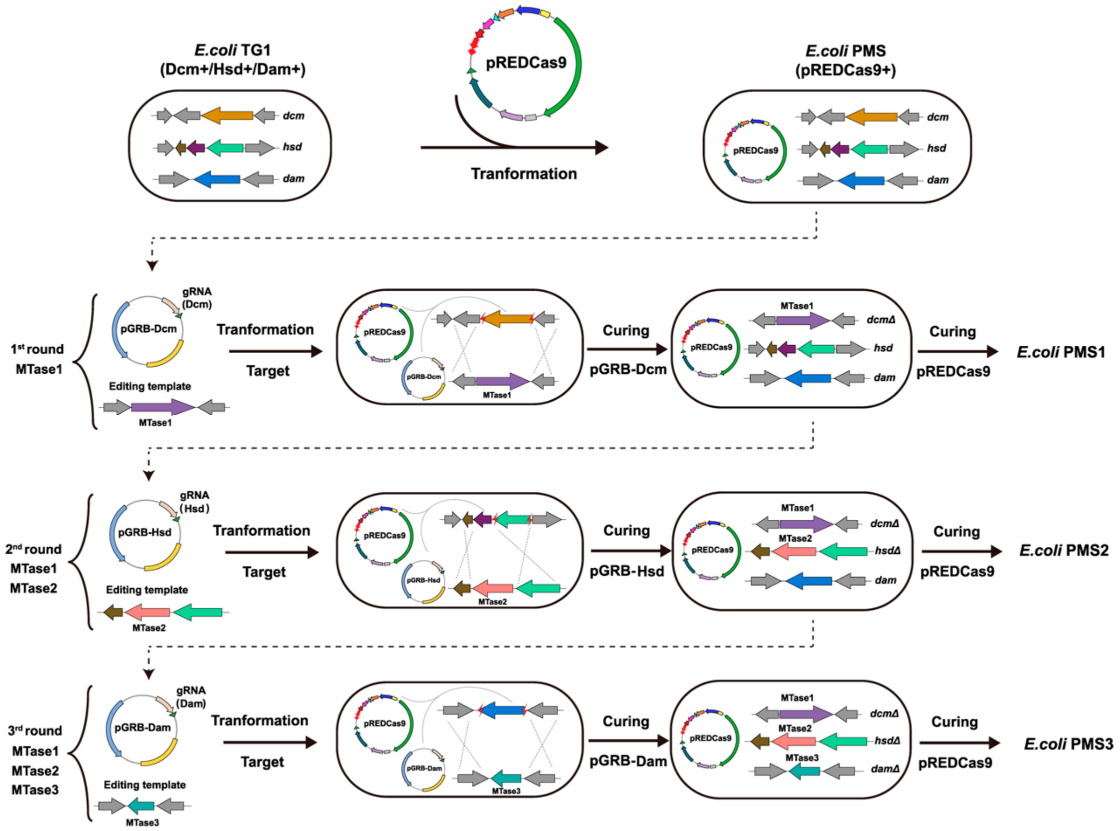

Figure S1. Schematic representation of PMS tools construction. The CRISPR-Cas9 based system involves co-transformation of editing template and gRNA plasmid into cells that express Cas9 and the λ-Red recombinase system. This approach enables gene replacements that allow the cells to evade CRISPR-mediated cutting by eliminating both the protospacer and PAM sequences. Additionally, when induced with arabinose, the gRNA targeting the ori region is expressed to eliminate plasmid pGRB from the cells.

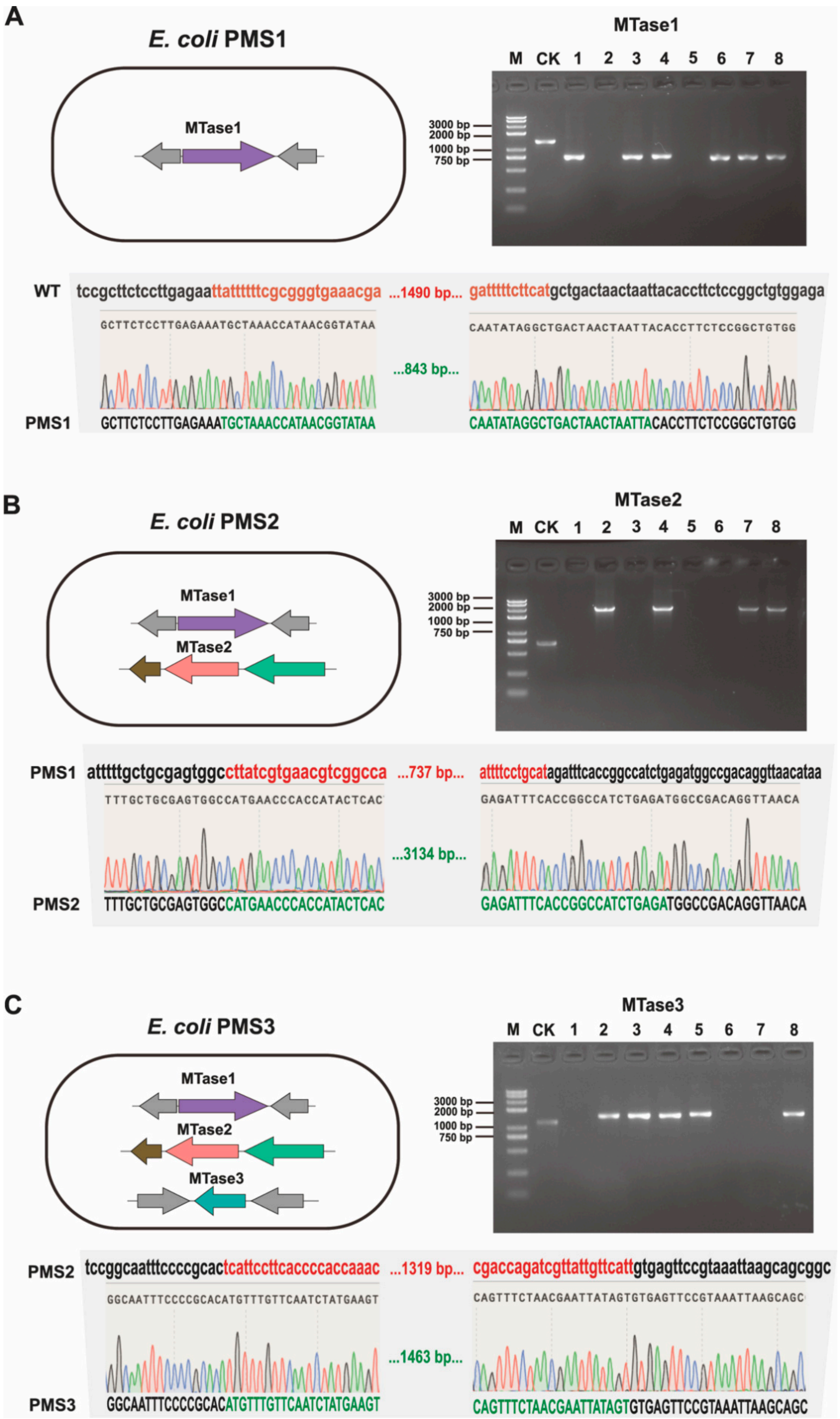

Figure S2. Development, verification and sequencing analysis of PMS tools. (A) The *hsdM* gene (1490 bp) of the *E. coli* W3110 strain was replaced by *mtase1* gene using the pRedCas9 system. (B) The *dam* gene (737 bp) of the *E. coli* PMS1 strain was replaced by *mtase2* gene using the pRedCas9 system. (C) The *dcm* gene (1319 bp) of the *E. coli* PMS2 strain was replaced by *mtase3* gene using the pRedCas9 system. “M” lane, the standard DNA marker. “CK” lane, the PCR product from the wild-type strain as a control.

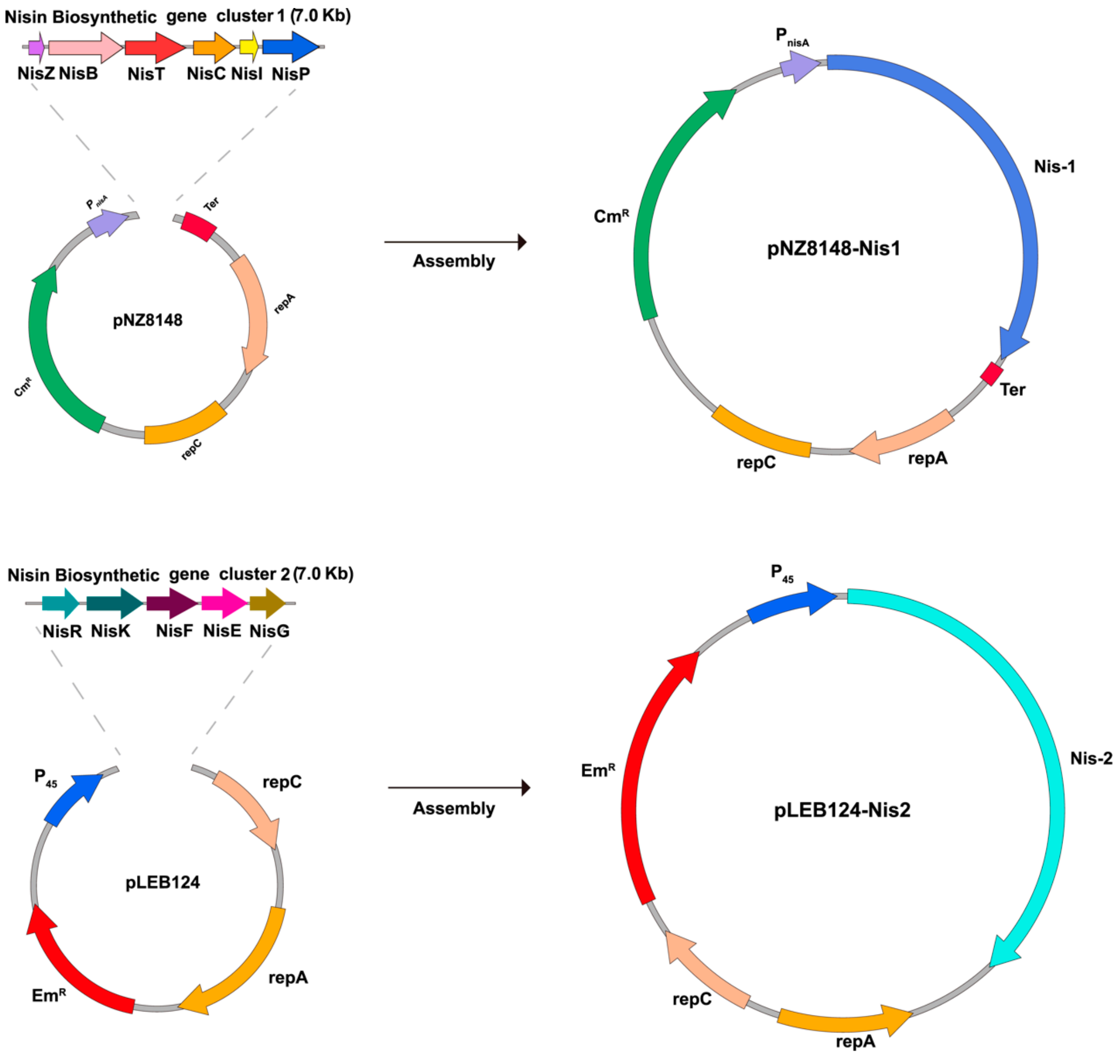

Figure S3. The workflow of constructing pNZ8148-Nis1 and pLEB124-Nis2 plasmids. The Nis1 and Nis2 gene clusters were successfully cloned from the genome via PCR method. Following this, the vectors pNZ8148 and pLEB124 were linearized using PCR. The Nis1 and Nis2 fragments were then assembled with the respective linearized vectors using seamless cloning, resulting in the generation of the plasmids pNZ8148-Nis1 and pLEB124-Nis2.

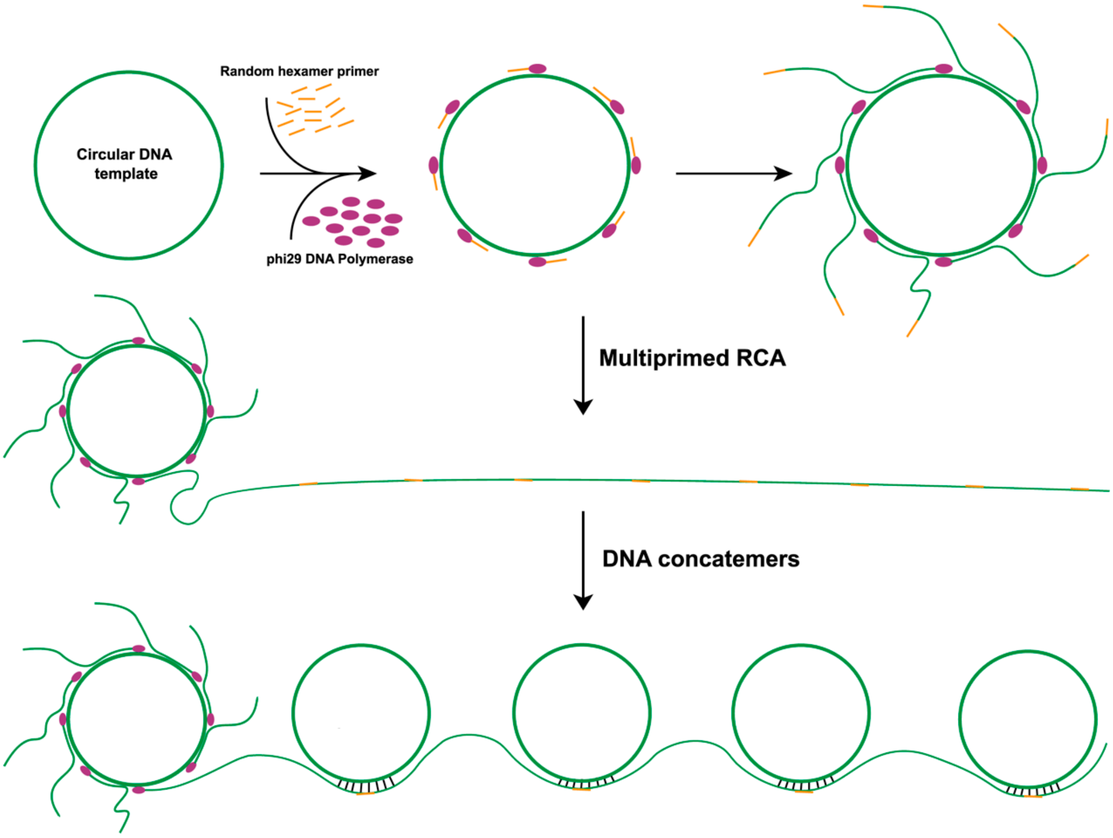

Figure S4. Schematic representation of concatomeric RCA products of plasmid. In RCA, the utilization of multiple primers capable of hybridizing with a single circular template enables the initiation of multiple amplification events, resulting in the generation of multiple RCA products (Multiprimed RCA). The RCA product comprises a concatemer consisting of numerous tandem repeats complementary to the circular template, ranging from tens to hundreds in number.

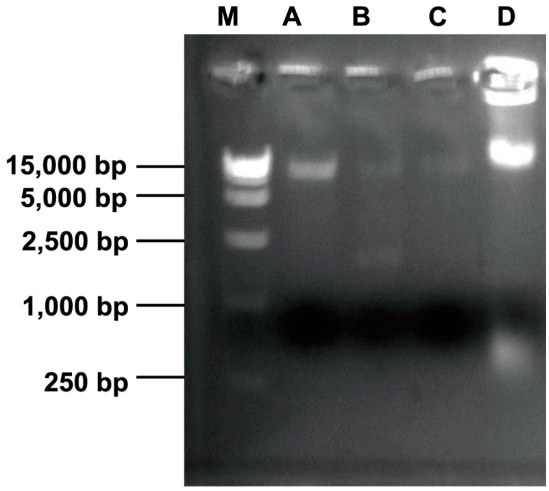

Figure S5. The DNA gel electrophoresis image of RCA-PMS products of various plasmids. M, DL15000 DNA marker; A, RCA-PMS product of pNZ8148; B, RCA-PMS product of pLEB124; C, RCA-PMS product of pNZTS-Cas9; D, RCA-PMS product of pNZTS-cBE.

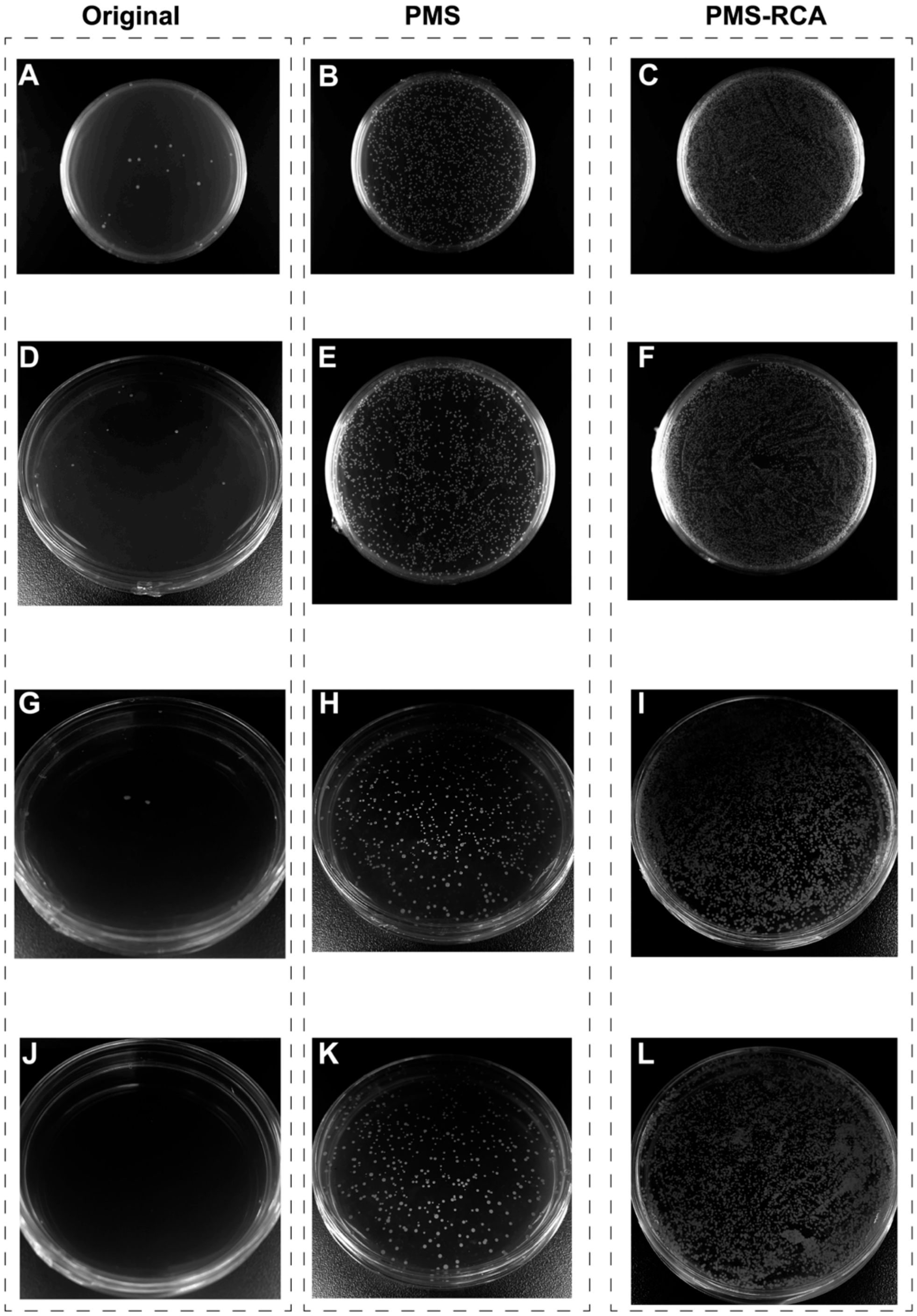

Figure S6. Representation of transformation efficiency on agar plates. (A) Transformants of *L. lactis* with original pNZ8148. (B) Transformants of *L. lactis* with PMS-modified pNZ8148. (C) Transformants of *L. lactis* with PMS-RCA-modified pNZ8148. (D) Transformants of *L. lactis* with original pLEB124. (E) Transformants of *L. lactis* with PMS-modified pLEB124. (F) Transformants of *L. lactis* with PMS-RCA-modified pLEB124. (G) Transformants of *L. lactis* with original pNZTS-Cas9. (H) Transformants of *L. lactis* with PMS-modified pNZTS-Cas9. (I) Transformants of *L. lactis* with PMS-RCA-modified pNZTS-Cas9. (J) Transformants of *L. lactis* with original pNZTS-cBE. (K) Transformants of *L. lactis* with PMS-modified pNZTS-cBE. (L) Transformants of *L. lactis* with PMS-RCA-modified pNZTS-cBE.

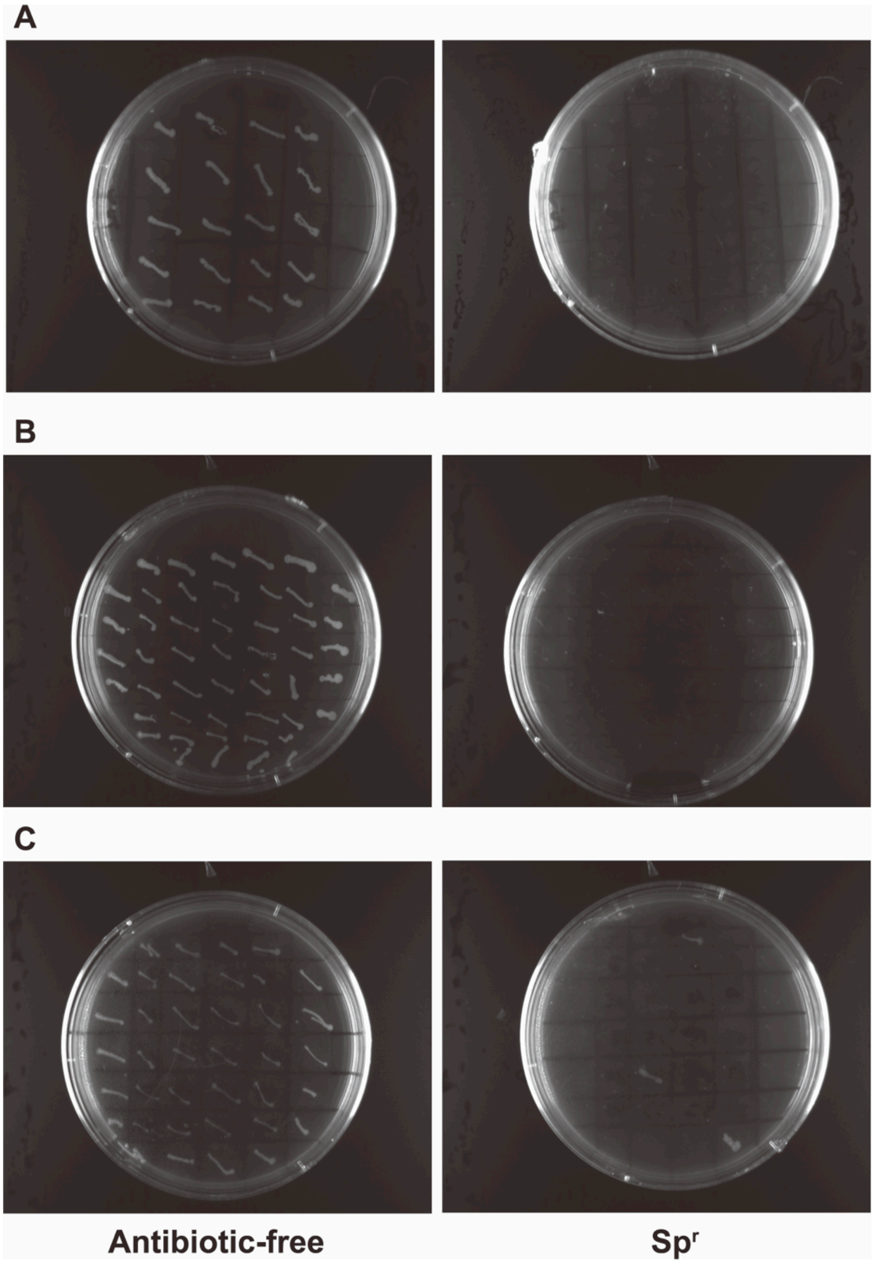

Figure S7. The plasmid curing of pRedCas9 for engineered *E. coli* PMS1 (A), *E. coli* PMS2 (B) and *E. coli* PMS3 (C). The pRedCas9 plasmids were cured by incubating the cell cultures in antibiotic-free LB medium at 42 °C, followed by streaking onto LB agar plates incubated at 37 °C. Cells that successfully lost the plasmid were able to grow on antibiotic-free plates (left) but failed to grow on plates containing Spectinomycin (right). Spr, Spectinomycin.

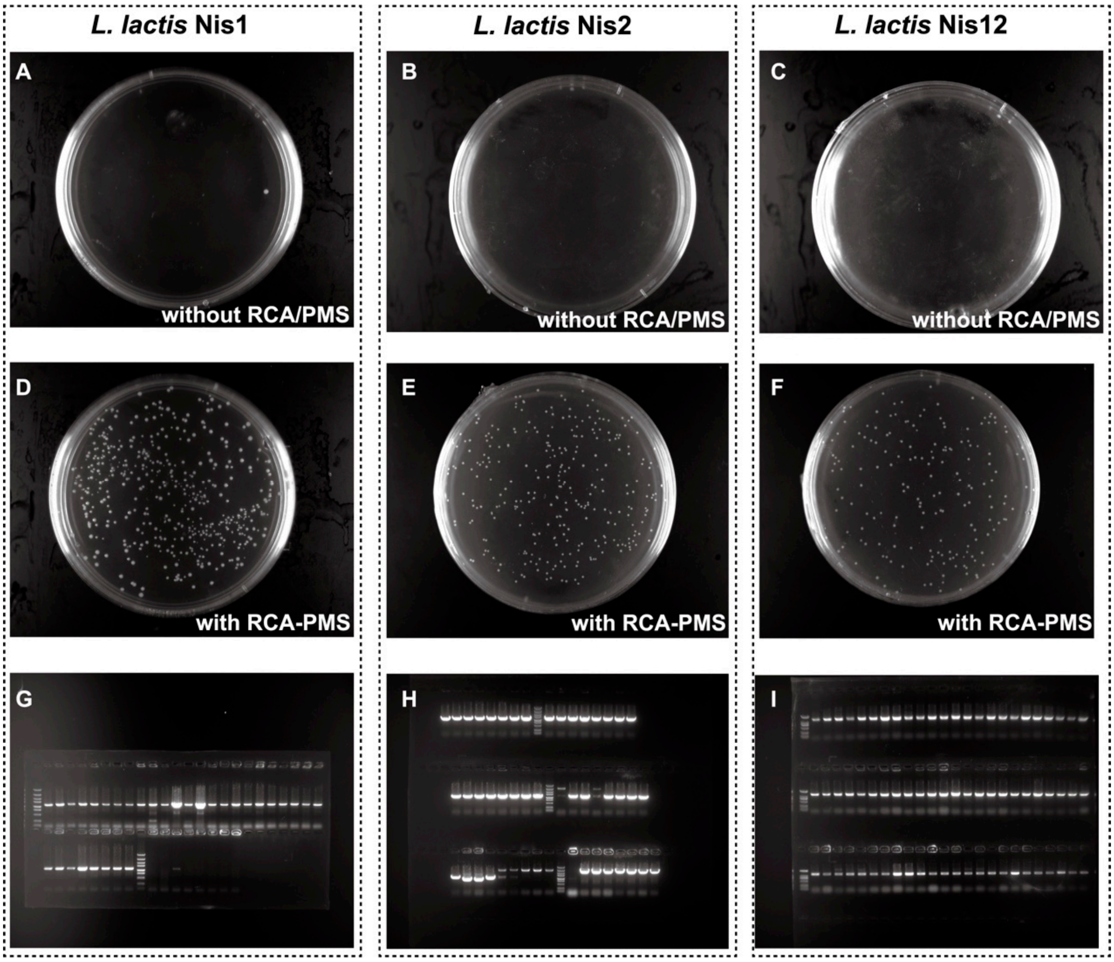

Figure S8. Transformation efficiency and verification of Nisin biosynthesis gene clusters. (A) Transformation efficiency for Nis-1 gene cluster without RCA/PMS. (B) Transformation efficiency for Nis-2 gene cluster without RCA/PMS. (C) Transformation efficiency for complete Nisin biosynthesis gene cluster without RCA/PMS. (D) Transformation efficiency for Nis-1 gene cluster with RCA-PMS. (E) Transformation efficiency for Nis-2 gene cluster with RCA-PMS. (F) Transformation efficiency for complete Nisin biosynthesis gene cluster with RCA-PMS. (G) Verification for *L. lactis* Nis1 by colony PCR. (H) Verification for *L. lactis* Nis2 by colony PCR. (I) Verification for *L. lactis* Nis12 by colony PCR.

REFERENCES

1. Tian, K.; Hong, X.; Guo, M.; Li, Y.; Wu, H.; Caiyin, Q.; Qiao, J. Development of Base Editors for Simultaneously Editing Multiple Loci in *Lactococcus Lactis*. *ACS Synth. Biol.* **2022**, acssynbio.1c00561, doi:10.1021/acssynbio.1c00561.
2. Li, Y.; Lin, Z.; Huang, C.; Zhang, Y.; Wang, Z.; Tang, Y.; Chen, T.; Zhao, X. Metabolic Engineering of Escherichia Coli Using CRISPR–Cas9 Meditated Genome Editing. *Metab. Eng.* **2015**, *31*, 13–21, doi:10.1016/j.ymben.2015.06.006.
